# Validation of AQoL-8D: a health-related quality of life questionnaire for adult patients referred for otolaryngology

**DOI:** 10.1007/s00405-021-06689-6

**Published:** 2021-02-25

**Authors:** Anita Obrycka, Jose-Luis Padilla, Artur Lorens, Piotr Henryk Skarzynski, Henryk Skarzynski

**Affiliations:** 1 World Hearing Center, Institute of Physiology and Pathology of Hearing, ul. Mokra 17, Kajetany, Nadarzyn, 05-830 Warsaw, Poland; 2 Mind, Brain and Research Centre, Dept. Methodology of Behavioral Sciences, School of Psychology, University of Granada, Granada, Spain; 3 Heart Failure and Cardiac Rehabilitation, Department of the Medical, University of Warsaw, Warsaw, Poland; 4 Institute of Sensory Organs, Kajetany, Poland

**Keywords:** Health-related quality of life, Validation, Patient-reported outcome measures, Otolaryngology, Hearing impairment

## Abstract

**Purpose:**

The purpose of the study was to validate the AQoL-8D questionnaire in the adult population of patients referred to an otolaryngology clinic.

**Methods:**

AQoL-8D was translated into Polish. 463 patients (age18–80 years) with otolaryngological conditions were assessed with the AQoL-8D, SF-6D, and SWLS questionnaires. We investigated the item content-relevance, factor structure by means of Confirmatory Factor Analysis, corrected item-total correlations, Cronbach’s alpha, Pearson correlation of the AQoL-8D scores with results from SF-6D and from the SWLS questionnaires. Finally, ANOVA was used to test the AQoL-8D ability to group the HRQoL of patients in terms of their otolaryngological management type.

**Results:**

The median score of item content-relevance was 5.0 for all AQoL-8D items. Confirmatory Factor Analysis revealed the following fit indices: Comparative Fit Index = 0.81; Tucker–Lewis Index = 0.80; and Root Mean Square Error of Approximation = 0.07. Cronbach's alpha for AQoL-8D dimensions ranged from 0.48 to 0.79. Mean item-total correlations over all dimensions, super dimensions, and the instrument overall were higher than 0.3. There was a significant Pearson correlation between the results obtained with AQoL-8D and SF-6D (*r* = 0.68), and with AQoL-8D and SWLS (*r* = 0.43). A one-way ANOVA showed a significant effect of management type on HRQoL as measured by AQoL-8D [*F*(4,458) = 6.12, *p* < 0.001]

**Conclusion:**

AQoL-8D provides valid and reliable measures of HRQoL in patients undergoing otolaryngological treatment. Because it is a generic questionnaire, it is possible to make general comparisons of otolaryngology outcomes with those from other subspecialties.

## Introduction

To make comparisons between one treatment or health problem with another, it is recommended in principle to use standardised Health-Related Quality of Life (HRQoL) questionnaires which are validated, generic (non-disease-specific), and can provide a single outcome measure—a Utility Index (UI) [1, 2]. In comparison to other subspecialties, there is a relative lack of such utility analyses in otolaryngology [3]. So far, HRQoL has been evaluated mainly in the specific domains of hearing aids, cochlear implants, and head and neck cancer using a wide range of instruments, mostly disease-specific [4].

Evaluation of the general population of patients referred to otolaryngology clinics using a generic HRQoL questionnaire would be particularly useful, as the outcomes thus obtained would allow broader comparisons of otolaryngology with other subspecialties, especially since the majority of otolaryngology problems are neither life-threatening nor require surgery and so do not allow comparisons in terms of mortality rate or surgical success rate.

The only study to date on HRQoL in a population referred to an otolaryngology clinic was performed by Swan et al. [5] on a group of 9005 adult patients. The Health Utilities Index mark 3 (HUI-3) instrument was applied before and after treatment. The authors found that patients treated surgically or with hearing aids reported a small but significant improvement in their HRQoL, while patients treated in other ways reported no significant improvement [5]. These results suggest that HUI-3 is probably not sensitive enough to measure changes in HRQoL in all groups of otolaryngology patients.

From the published findings it is not clear what instrument could capture HRQoL for a population undergoing an otolaryngological intervention [5–7]. Nevertheless, the choice of HRQoL instrument is important and needs to be made according to the target population. Methods for HRQoL measurement vary not only in terms of the scaling technique and the model used to derive the scoring formula, but also in terms of the type and scope of the questions, the number of health states measured, and the number of dimensions covered [8, 9]. Because there are no single generally agreed-upon definition of HRQoL and no gold standard for HRQoL measurements, the general recommendation is to use an instrument that covers all the dimensions considered important for the study population [1, 10].

Moreover, since each instrument differs in its sensitivity to particular health dimensions, the question arises as to what instrument would provide valid measures for the population of patients referred to otolaryngology, taking into account diversity of this population in terms of age, type of intervention, and health status. In trying to answer this question, it is important to realise that many otolaryngology problems (e.g. hearing loss, vertigo, tinnitus) are often associated with long-lasting disabilities which usually have severe psycho-social consequences [11–14]. Therefore, a HRQoL instrument suitable for evaluating otolaryngology patients needs to place a strong emphasis on items in the psycho-social domain. An analysis of the descriptive systems of most common instruments, done by Richardson et al. [15], showed that each instrument displayed significant differences in the proportion of items related to the physical and psycho-social domains. In their analysis authors compared five generic health utility measures: EuroQol-5 Dimension (EQ-5D), Health Utilities Index mark 3 (HUI-3), 15-dimensional measure of HRQoL (15D), Short-Form Six-Dimension (SF-6D), and Assessment of Quality of Life-8 Dimensions (AQoL-8D). AQoL-8D is the instrument which has items related to hearing and with the largest proportion of items in the psycho-social domain, therefore, is mostly suitable to capture domains important for otolaryngology.

The AQoL-8D consists of 35 items covering 8 dimensions: Independent Living, Pain, Senses, Mental Health, Happiness, Coping, Relationships, and Self-worth [16, 17]. To ensure its content validity (the extent to which an instrument covers all aspects of the intended construct, that is, HRQoL), AQoL-8D was derived using psychometric methods. For each dimension, each health state has been evaluated using both a visual analogue sale (VAS) and a time trade-off (TTO) technique, which are common methods used to estimate the utility of the health state. To derive a single valid utility index (UI), a two-stage multiplicative–econometric scaling procedure was used [18].

The general aim of this research is to obtain valid evidence of the AQoL-8D questionnaire in a population of adult patients referred to our otolaryngology clinic.

## Material and methods

### Participants

All adult patients who were referred during 3 consecutive months for a consultation with the Institute of Physiology and Pathology of Hearing (IPPH) in Warsaw were asked to participate in the study. A total of 463 patients consented to take part.

### Study design

Between 01.08.2016 and 31.10.2016 patients were evaluated with a translated Polish version of the AQoL-8D questionnaire; a Polish adapted version of the SF-36 (a HRQoL assessment questionnaire) [19–21]; an adapted version of the SWLS (Satisfaction with Life Scale, an assessment of subjective well-being) [22, 23]; and asked general survey questions on socio-demographic variables. All scales and sociodemographic questions were compiled in a single survey questionnaire. Once patients were told about the research project’s aims and asked to take part in the study, they filled out the consent form and questionnaire. Completed questionnaires were put in a plain envelope to guarantee confidentiality. The study was designed and conducted according to the Declaration of Helsinki and the study protocol was approved by the Institutional Review Board IFPS: KB/06 /2016.

### Translation of the AQoL-8D into Polish

To validate the AQoL-8D in the target population (patients referred to otolaryngology clinic), the translation of the AQoL-8D into Polish was conducted. A “committee approach to translation” design was applied to develop the Polish version of the AQoL-8D following the TRAPD (Translation, Review, Adjudication, Pretesting, and Documentation) model [24]. A “committee-approach to translation” is a three-stage translation method intended to provide an appropriate translated version of the questionnaire in the target language considering linguistic, cultural and content issues. This design is recommended in different professional guidelines, for instance, the International Test Commission Guidelines for Translating and Adapting Test [25]. The translation team consisted of two translators, a reviewer, and an adjudicator. At the first stage of the process, the translators worked separately, preparing independent translations. The reviewer’s task in the team was to review and edit the translations of AQoL-8D items, instructions, and rubrics. Next, the translation team led by the adjudicator met and discussed the two translations item by item until they arrived at the draft of the Polish version of the AQoL-8D questionnaire. The adjudicator took care of methodological issues through the whole translation process and documented all points discussed during the meetings.

To examine the quality of translation, the comparability between the original and Polish versions of the AQoL-8D questionnaire was investigated by an appraisal by a panel of experts. Thirteen experts were asked to rate each item by the comparability of the original version and the Polish version of the AQoL-8D questionnaire on a numbered scale from 1 (no comparability) to 5 (perfect comparability). For all AQoL-8D items, the median score of comparability was 5.0, with the means ranging from 4.3 to 5.0. These results show that experts assessed all Polish items in a highly comparable way to that of the original version. Moreover, the inter-quartile ranges of experts’ ratings were never above 1, indicating a high level of agreement among the experts. The results of experts’ appraisal demonstrate the high quality of AQoL-8D translation into Polish.

### Data analysis

The first step in researching the AQoL-8D’s validity in a population of patients referred to otolaryngology clinic was to examine item content-relevance by an expert appraisal. Thirteen experts were recruited from IPPH employees working in different departments of the Institute. The group consisted of medical doctors (specialists in otolaryngology, audiology, and phoniatrics), audiologists, speech-language pathologist, and psychologists. Experts were asked to rate the relevance of each AQoL-8D item on a numbered scale from 1 (highly irrelevant) to 5 (highly relevant). If the rating was below 4, they were asked to provide comments explaining why in their opinion the item content is not relevant for the target population.

The second validation step was to examine the AQoL-8D questionnaire’s internal structure, understood as its factor structure and score reliability. Confirmatory Factor Analysis (CFA) was performed to examine whether the data fitted the hypothesised eight-dimensional model of the original version of AQol-8D [26]. The AQoL-8D consists of 35 items covering 8 dimensions: Independent Living, Pain, Senses, Mental Health, Happiness, Coping, Relationships, and Self-worth. Three of them (Independent Living, Pain, Senses) are related to a physical ‘super-dimension’ and the remaining five to a psycho-social ‘super-dimension’ [16, 17].

Calculations were done of standardised factor loadings as well as the usual fit indices (CFI, Comparative Fit Index; TLI, Tucker–Lewis Index; and RMSEA, Root Mean Square Error of Approximation). A Weighted Least Square Mean and Variance-adjusted (WLSMV) estimation method was applied which considered all AQol-8D items as categorical variables. Next the item analysis was done and descriptive statistics (mean and standard deviation) calculated. Corrected item-total correlations were assessed to examine the discrimination of the AQoL-8D items in all dimensions, in the super dimensions, and for the whole instrument. Cronbach alpha coefficients were computed to examine the reliability of the AQoL-8D scores.

The third step in obtaining validity evidence for AQoL-8D was to analyse the Pearson correlations of the UI of AQoL-8D with the UI of SF-6D (as calculated from SF-36). A strong correlation between those two measures indicates that they are both gauging the same construct—HRQoL. In addition, the correlation between the AQoL-8D UI and the SWLS total score was calculated to examine the relationship of AQoL-8D results to a measure of subjective well-being (which is a concept similar to HRQoL). The Pearson correlation of the AQoL-8D UI with the SF-36 dimensions was also calculated to investigate the hypothesis that psycho-social domains were well represented in the AQoL-8D total score.

Finally, the ability of AQoL-8D to differentiate between groups of patients classified into different management types was examined. One-way ANOVA was used to determine whether the AQoL-8D UI and the results of the AQoL-8D dimensions and the super dimensions differed significantly between management types. Statistical analyses were performed using MPlus version 7.3 and Statistica version 12.0.

## Results

### Participants

A total of 463 patients aged between 18 and 80 years old participated in the study. Their mean age was 47 years old (SD 16.2); 201 were men aged between 18 and 76 years (mean 47; SD 16.7), and 262 were women, aged 18–80 years old (mean 47; SD 15.8). Age and gender of the study group are presented in Table 1. Patients were classified into five types of otolaryngological management: 13% medical treatment (medication); 22% cochlear implantation (CI); 12% hearing aid (HA) or middle ear implant (MEI) provision; 44% surgery (other than CI and MEI); and 9% given reassurance or advice on self-management (the ‘reassure’ group).

**Table 1:** Age and gender of the study group

Age range	Male		Female		Total	
	*N*	%	*N*	%	*N*	%
18–20	14	7.0	9	3.4	23	5.0
21–30	34	16.9	48	18.3	82	17.7
31–40	21	10.4	37	14.1	58	12.5
41–50	43	21.4	47	17.9	90	19.4
51–60	38	18.9	63	24.0	101	21.8
61–70	38	18.9	41	15.6	79	17.1
71–80	13	6.5	17	6.5	30	6.5

### Item content-relevance

For all AQoL-8D items, the median score of content-relevance was 5.0, with the means ranging from 4.1 to 5.0. The lowest mean experts’ ratings were obtained for items related to vision, 4.1 (item 28); mobility, 4.2 (item 15); and degree of pain, 4.5 (item 22). These results show that experts assessed most AQoL-8D items as highly relevant to measure HRQoL in patients who are receiving otolaryngological treatment. Moreover, the inter-quartile ranges of experts’ ratings were zero for all AQoL-8D items except for items no. 28, 15, and 22 where the inter-quartile ranges were 2, 1, and 2, respectively.

### Internal structure

**Fig. 1:**
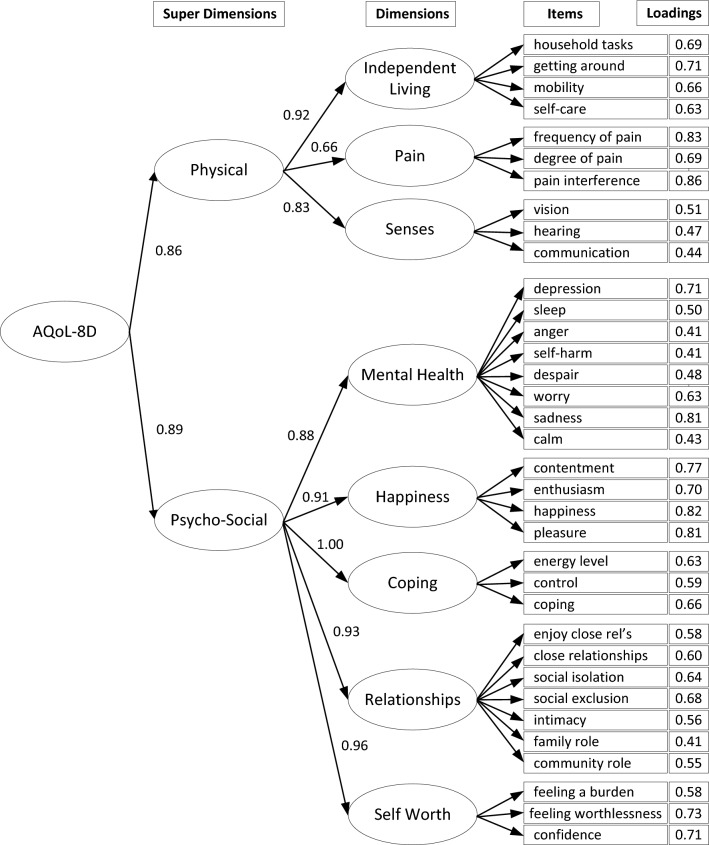
Structure of AQoL-8D and the factor loadings on super dimensions, dimensions, and individual items

CFA was also used to test the original eight-dimension model for the AQoL-8D. The model, together with the standardised factor loadings for items, dimensions, and super dimensions, is presented in Fig. 1. The values of the fit statistics were: CFI = 0.85, TLI = 0.84, and RMSEA = 0.07. While the values of CFI and TLI were slightly below the usual cut-off [27], the RMSEA values were considered good.

Table 2 shows the mean total scores, standard deviations, Cronbach's alpha, and item discriminations for the 8 dimensions, 2 super dimensions, and the whole instrument. Values of Cronbach's alpha for the AQoL-8D dimensions ranged from 0.48 (Senses) to 0.79 (Happiness) and exceeded 0.7 for the super dimensions and the whole instrument. Mean item-total correlations for all dimensions, super dimensions, and instrument were higher than 0.3. The lowest individual item-total correlation was 0.19 for item 28 (related to vision).

**Table 2:** Descriptive statistics of AQoL-8D scores; discrimination capacity of the items; and scores reliability for the dimensions, super dimensions, and the overall instrument

	Descriptive statistics		Discrimination capacity of items			Scores reliability
	Mean	SD	Mean item-total correlation	Min item-total correlation	Max item-total correlation	Cronbach’s alpha
*Dimensions*						
Independent living	0.89	0.11	0.45	0.34	0.50	0.64
Pain	0.78	0.21	0.62	0.59	0.65	0.78
Senses	0.74	0.16	0.31	0.19	0.46	0.48
Mental health	0.58	0.11	0.48	0.35	0.62	0.77
Happiness	0.76	0.13	0.61	0.47	0.69	0.79
Coping	0.80	0.13	0.37	0.35	0.40	0.56
Relationships	0.73	0.14	0.45	0.31	0.59	0.74
Self-worth	0.81	0.13	0.49	0.45	0.53	0.67
*Super dimensions*						
Physical	0.65	0.18	0.45	0.34	0.55	0.77
Psycho-social	0.38	0.16	0.54	0.29	0.70	0.92
*Instrument*						
AQoL-8D UI	0.68	0.17	0.50	0.28	0.67	0.93

### Relation to other variables

**Fig. 2:**
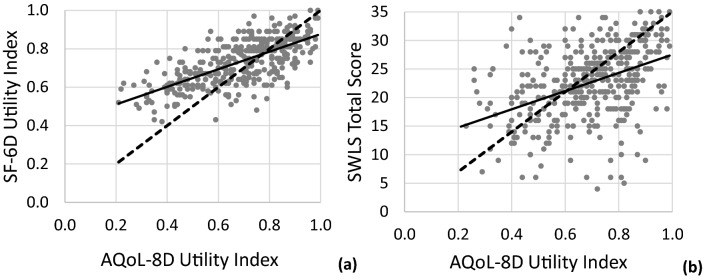
Scatterplots of **a** SF 6D vs AQoL-8D utility scores; **b** SWLS total scores vs AQoL-8D utility scores. Solid lines are regressions; dashed lines are theoretically ideal 1:1 relationships

Figure 2a shows that the Pearson correlation between UIs calculated from SF-6D and AQoL-8D was strong (*r* = 0.68) and significant (*p* < 0.01). Figure 2b shows that there was a smaller but significant (*p* < 0.01) correlation between AQoL-8D scores and the SWLS instrument (*r* = 0.43). The correlations of AQoL-8D UI with SF-36 dimensions were all significant at the level of 0.01 and were as follow: 0.42, general health; 0.51, bodily pain; 0.44, physical functioning; 0.45, role limitation (physical); 0.56, vitality; 0.52, social functioning; 0.42, role limitation (emotional); 0.62, mental health; 0.59, PCS (Physical Component Summary); 0.68, MCS (Mental Component Summary).

A one-way ANOVA showed a significant effect of management type on UI measured by AQoL-8D [*F*(4,458) = 6.12, *p* < 0.001]. Similarly, the significant effect of management type on HRQoL measured in the Physical super dimension [*F*(4, 458) = 8.22, *p* < 0.001] and in the Psycho-social super dimension [*F*(4, 458) = 3.65, *p* = 0.006] was observed. Moreover, a significant effect of patient management type on HRQoL was found in five out of eight dimensions: Independent Living, *F*(4,449) = 3.76, *p* = 0.005; Senses, *F*(4,435) = 18.49, *p* < 0.001; Happiness, *F*(4,441) = 3.87, *p* = 0.004; Relationships, *F*(4,447) = 2.85, *p* = 0.024; and Self-worth, *F*(4,439) = 3.99, *p* = 0.003. The effect was not observed for Pain [*F*(4,431) = 1.25, *p* = 0.291]; Mental Health [*F*(4,434) = 1.97, *p* = 0.098]; or Coping [*F*(4,450) = 2.27, *p* = 0.061]. The results of AQoL-8D UI obtained in patients assigned to each of the five groups of otolaryngological management, including post hoc comparisons, are shown in Table 3.

**Table 3:** ANOVA comparisons of AQoL-8D UI results for five types of otolaryngological management

Management type	*N*	Mean	SD	Post hoc comparisons			
				Medication	CI	HA or MEI	Surgery
Medication	61	0.74	0.16				
CI	103	0.64	0.17	< 0.001			
HA or MEI	54	0.62	0.17	< 0.001	0.484		
Surgery	204	0.70	0.16	0.081	0.005	0.003	
Reassure	41	0.71	0.15	0.418	0.022	0.009	0.603

## Discussion

To reliably interpret any HRQoL measure, it must have strong validity, and this property is currently considered the most important metric [28–30]. Following the current understanding of validity, "validation" is considered to be the ongoing endeavour in which different strands of validity evidence are gathered, summarised, and integrated to support the intended interpretation of the selected HRQoL measure [31]. Among the sources of validity evidence, i.e., validation methods, two approaches are particularly relevant for HRQoL measures: “internal structure” and “relation to other variables” [32]. The rationale behind the focus on the internal structure of an instrument is to test whether the data fit a hypothetical measurement model of how items and dimensions account for responses. “Relation to other variables” can be explored in three ways: the extent to which a measure of the intended concept correlates with another widely accepted measure of the same concept (i.e. traditional “convergent validity”); correlation with other measures of theoretically related constructs; and by demonstrating when a questionnaire can discriminate between two groups known to differ on the variable of interest. All these strands of validity evidence can support the interpretation of an HRQoL measure in a target population.

The assessment of item content-relevance shows that experts regarded almost all of the AQoL-8D items as highly relevant for measuring HRQoL in patients receiving otolaryngological treatment. The results also indicate a high level of agreement among the experts. The expert appraisal revealed that only 3 out of 35 AQoL-8D items had a content-relevance which raised doubts among experts about their importance. Only item 28 relating to vision (Senses dimension), item 15 with a reference to mobility (Independent Living dimension), and item 22 considering the degree of pain (Pain dimension) were rated less relevant than the rest of the items. Moreover, for those same items the inter-quartile ranges of the experts ratings were the highest, showing that there were appreciable differences in the experts’ opinions. For the target population—patients referred for otolaryngological treatment—pain and mobility problems are not frequent, although some diseases (e.g., otitis media) can cause pain and some (e.g., vertigo) can result in mobility problems. The question about vision seemed to be the least relevant for the assessed population.

Results of the CFA allow us to be confident that the data obtained from the patients responding to the Polish version of AQoL-8D can be accounted for by the same model as the original version. The results of the CFA of the original version done by Richardson et al. [18] revealed almost identical factor loadings as the ones of the Polish version presented in Fig. 1. Some values of the fit statistics were below the usual cut-offs [27]. To interpret these results, differences between the validation study of the original AQoL-8D and the validation study of the Polish version should be taken into account. The validation of the original AQoL-8D was performed in a sample of the general population, while the Polish version was validated in a population of patients undergoing otolaryngological treatment. In addition, there are also differences in administration modes used in both validation studies. It seems that the different CFA results might be explained by sample dependency, the difference in administration mode, translation effects, and the special characteristics of patients undergoing otolaryngological treatment.

Values of Cronbach's alpha above 0.7 are generally considered adequate [33]. In the current study, the highest values of Cronbach's alpha were found for AQoL-8D and for the Psycho-Social super dimension (above 0.9). Values below 0.7 were obtained for four dimensions: Independent Living, Senses, Coping, and Self-worth. This is in line with the finding that Cronbach's alpha depends on the length of the test [34, 35]. In the case of the AQoL-8D questionnaire, both the instrument itself and the Psycho-Social super dimension comprise a large number of items (35 and 25, respectively). In contrast, dimensions with the lowest values of Cronbach's alpha consist of just 3 or 4 items for each dimension. A value below 0.7 for the Senses dimension has also been reported by the authors of AQoL-8D [18, 36]; they hypothesised that this complex dimension consists of too few items to be a good stand-alone scale.

Mean item-total correlations for all dimensions were higher than 0.30, indicating that the items had a high discrimination capacity. The lowest item-total correlation (0.19) was obtained for item number 28 relating to vision (in the Senses dimension). This is not surprising, since the HRQoL was assessed in a group of patients receiving otolaryngological treatment, not the general population. Moreover, the vision question received the lowest rating in terms of relevance.

The strong correlation between the AQoL-8D UI and the SF 6D UI confirms that both instruments measure the same construct (that is, HRQoL). The value of our correlation coefficient (*r* = 0.68) is in the same range as reported by other studies (0.55–0.81) which evaluated HRQoL in both healthy subjects and in patients with different health conditions (arthritis, asthma, cancer, chronic obstructive pulmonary disease, depression, diabetes, chronic heart disease, stroke, hearing problems) [37–41]. The correlation between the AQoL-8D UI and the SWLS total score was lower (by 0.25) than between AQoL-8D and SF-6D. This weaker correlation is not surprising since those instruments are not measuring the same construct as examined here but a closely related concept instead (HRQoL in the first case and subjective well-being in the second). Lower correlation coefficients between AQoL-8D and SWLS (0.44–0.73) were also reported in a series of reports by Richardson et al. [37–40].

Analysing the relation between AQoL-8D UI and SF 6D UI (Fig. 2a) it is clear that AQoL-8D provided a greater range of scores (0.21–0.99) in the tested population compared to SF-6D (0.42–1.00). The utility scores achieved by SF-6D are compressed into the upper range of the utility scale. That is, a change in scores reported by SF-6D corresponds to a much larger change in scores in AQoL-8D. The outcome is that the regression line has a shallower slope compared to the theoretically ideal relationship. This indicates that AQoL-8D has a greater capacity to detect changes in the HRQoL of the target population compared to the SF-6D instrument. Moreover, AQoL-8D has the highest correlation with the Mental Health dimension and with the Mental Component Summary of SF-36. This is in line with findings reported by Richardson et al. [36] and confirms the substantial effect of the psycho-social component of AQoL-8D in HRQoL assessments.

We measured the HRQoL of a broad spectrum of patients referred to our otolaryngology clinic. To enlarge the evidence base for validity, relations to other variables were examined—the group differences in AQoL-8D score between management types. This approach also called the “known group method”, is a common way to enlarge the scope of validity evidence [42]. After classifying our patients into five types of otolaryngological management, we found that the AQoL-8D scores differed substantially across management type (from 0.62 for those provided with a hearing aid to 0.74 for those treated with medication). The mean value for the comparison population was 0.80, indicating that the overall HRQoL of our otolaryngological sample was substantially poorer [43].

AQoL-8D is based on the WHO definition of health, with a primary emphasis upon handicap (activity limitation and participation restriction) rather than just impairment. According to WHO, impairment is defined as a problem in body function or structure, such as a significant deviation or loss [44]. Activity is the execution of a task or action by an individual, whereas participation is involvement in a life situation. We surmise that management types such as surgery and medication (which cure illness by substantially reducing impairment and restore normal activity and participation) result in better HRQoL that the management types (hearing aids and cochlear implants) used primarily in for chronic conditions such as sensorineural hearing loss (SNHL)—which are strongly associated with handicap (activity limitation and participation restriction). In SNHL, an impairment (cochlear damage) has direct and immediate effects on most aspects of auditory function, including sensitivity, resolution, discrimination ability, and resistance to noise. Any deficit of function, which can only be partially overcome by a prosthetic device such as a hearing aid or cochlear implant, produces a deficit in activity, especially speech perception and oral communication. In turn, reduced activity seriously impacts participation.

As speculated, the AQoL-8D utility index was significantly higher in the group of patients who had surgery or were treated with medication than in the group of hearing aid or cochlear implant users. A similar pattern was also seen in AQoL-8D scores both in the Physical and Psycho-Social super dimensions. We have, therefore, demonstrated that AQoL-8D will return different scores for patient groups that vary in terms of management type.

Since HRQoL instruments produce different utility indexes [15], our results can only be applied with caution to the HUI-3 scores derived from patients having different otolaryngological managements. Swan et al. [5] have reported the following HUI-3 scores: 0.54 for those provided with hearing aids; 0.67 for those managed with medication; and 0.73 for those managed with surgery. The AQoL-8D scores for the same groups of patients stratified according to management type are 0.62, 0.74, and 0.70, respectively. That is, the results from both utility instruments are similar. Moreover, they show that the lowest HRQoL are for hearing aid users, which reflects the remaining handicap component (activity limitation and participation restriction) in that particular group. It would seem that the reason for this is that distortion, over and above simple attenuation, which accompanies SNHL is a prime factor in creating the difficulties such listeners experience in understanding speech (particularly in background noise). It follows that the amplification provided by hearing aids has only a limited capacity to return those abilities to normal [45].

The finding that the AQoL-8D is sensitive enough to demonstrate the negative impact of otolaryngology problems on quality of life is encouraging. As it is common practice to provide ‘population norms’: estimates of the average scores for different age-gender cohorts, future studies are needed with a special focus on elderly patients.

## Conclusions

Our results demonstrate that the AQoL-8D items are highly appropriate for measuring HRQoL in patients referred to otolaryngology clinics. They show that the patients’ responses to the items were highly consistent, and revealed that AQoL-8D can reliably distinguish various levels of HRQoL. We, therefore, conclude that AQoL-8D is a valid and reliable tool for assessing Health-Related Quality of Life in patients undergoing otolaryngological treatment.

## Data Availability

The data that support the findings of this study are available from the corresponding author, upon reasonable request. The data are not publicly available due to legal restrictions (their containing information that could compromise the privacy of research participants).
